# Bone Health in Former Artistic Gymnasts Aged 45 Years and Over: Case–Control Comparison with Controls and Reference Populations

**DOI:** 10.3390/ijerph23020159

**Published:** 2026-01-27

**Authors:** Patrícia Arruda de Albuquerque Farinatti, Cinthia Sousa, Rodrigo Zacca, Lurdes Ávila Carvalho, Jorge Mota, Igor Monteiro, Joana Carvalho, Nádia Souza Lima da Silva, Paulo Farinatti

**Affiliations:** 1 Graduate Program in Exercise and Sport Sciences, University of Rio de Janeiro State, Rio de Janeiro 20550-900, Brazil; patricia.farinatti@uerj.br (P.A.d.A.F.); cinthiasousa.radiologia@gmail.com (C.S.); igormont999@gmail.com (I.M.); nadialima@uerj.br (N.S.L.d.S.); 2 Research Center in Physical Activity, Health and Leisure (CIAFEL), Faculty of Sports, University of Porto, 4200-450 Porto, Portugal; rzacca@fade.up.pt (R.Z.); jmota@fade.up.pt (J.M.); jcarvalho@fade.up.pt (J.C.); 3 Laboratory for Integrative and Translational Research in Population Health (ITR), 4050-600 Porto, Portugal; 4 Laboratory of Sport Physiology, Faculty of Sports, University of Porto, 4200-450 Porto, Portugal; 5 Nucleus of Research in Human Motricity Sciences, Universidad Adventista de Chile, Chillán 3780000, Chile; 6 Research Center in Sports Sciences, Health Sciences and Human Development (CIDESD), University of Maia-UMAIA, 4475-690 Maia, Portugal; d012150@umaia.pt

**Keywords:** exercise, aging, bone health, bone, dual-energy X-ray absorptiometry, sports, health

## Abstract

**Highlights:**

**Public health relevance—How does this work relate to a public health issue?**

- Identifies how early-life participation in an osteogenic sport like artistic gymnastics can influence bone health decades later, a key factor in preventing osteoporosis;
- Addresses the burden of age-related bone loss by examining a population rarely studied: former gymnasts aged 45 years and over.

**Public health significance—Why is this work of significance to public health?**

- Demonstrates that former gymnasts have higher bone density and lower prevalence of osteopenia/osteoporosis than age-matched middle-aged and older adults;
- Supports the idea that maximizing bone accrual during growth can provide lasting protection and help preserve bone health in later in life.

**Public health implications—What are the key implications or messages for practitioners, policy makers, and/or researchers in public health?**

- Encouraging high-impact, weight-bearing exercise in childhood and adolescence may help establish a lifelong “bone reserve” and lower future osteoporosis risk;
- Highlights the need for lifespan-oriented bone health strategies that combine early mechanical loading benefits with targeted support in older adulthood, especially for postmenopausal females, who face accelerated bone loss despite earlier advantages.

**Abstract:**

Peak bone mass gained in youth is crucial for preventing osteoporosis. Artistic gymnastics (AG) is highly osteogenic, yet its long-term effects on adults ≥ 45 years are not well documented. This case–control study compared bone mineral density (BMD) and the prevalence of osteopenia/osteoporosis in former gymnasts, age-matched controls, and reference populations from Brazil and Portugal. Participants included 65 former gymnasts (32 males, 33 females; 45–84 years), who trained for 12.6 ± 4.3 years and included 41 international competitors, and 91 controls (37 males; 45–87 years). Whole-body and femoral BMD were assessed by DXA. Physical activity during youth (10–20 years) (PA-Youth) and the past decade (PA-10) was recorded. Reference data were drawn from large cohorts in Brazil (FIBRA, n = 828) and Portugal (CIAFEL, n = 1089). Former gymnasts had substantially higher PA-Youth than controls, while PA-10 was similar. Gymnasts displayed 4–6 times higher femoral Z-scores (neck and total) and a markedly lower prevalence of osteopenia/osteoporosis (males: 3% vs. 16%; females: 36% vs. 52%, *p* < 0.05). These benefits remained after adjustment for age, PA-10, and hormonal/calcium therapy. Relative to reference populations, gymnasts showed greater whole-body and femoral mineralization, with no osteoporosis cases (vs. 6–12% overall; 9–13% among those ≥60 years). Age-stratified analysis (45–59 and ≥60 years) revealed a consistently lower osteopenia prevalence across age groups, except in females ≥ 60 years. In conclusion, early-life AG participation is associated with enduring skeletal benefits, including higher bone mineralization and reduced osteopenia/osteoporosis in adults ≥ 45 years. The protective effect appears diminished in older females, likely reflecting prolonged postmenopausal bone loss.

## 1. Introduction

Osteoporosis, characterized by reduced bone strength and an increased risk of fractures, represents a major global public health concern [1,2]. According to World Health Organization criteria, osteoporosis is diagnosed when areal bone mineral density (BMD) is 2.5 standard deviations or more below the young adult reference mean (T-score ≤ −2.5), whereas osteopenia denotes an intermediate state of bone loss, defined by T-scores between −1.0 and −2.5 [2]. Although osteoporosis is most prevalent in older adults, its clinical expression is strongly influenced by early-life factors, particularly the acquisition of peak bone mass during growth. Evidence suggests that a 10% increase in peak bone mass achieved in youth may reduce fracture risk by up to 50% later in life [3,4].

Among modifiable factors influencing bone development, physical activity is particularly important [5,6]. Exercise enhances bone strength by applying mechanical loads through gravitational forces and muscle tension at tendon insertion points [7]. Numerous studies have shown that consistent physical activity during childhood and adolescence enhances bone mineral accumulation [4,6,8], with dynamic, weight-bearing activities providing the most significant site-specific improvements [4,6,9].

Artistic gymnastics imposes high dynamic mechanical loads on the skeleton up to 15 times body weight during jumps, landings, and acrobatic maneuvers, providing a potent stimulus for bone accrual during growth. This makes gymnastics an excellent model for studying long-term skeletal adaptations to weight-bearing physical activity [7,10,11]. The early exposure to gymnastics is linked to structural benefits in the proximal femur and distal radius (as higher trabecular density, and favorable bone geometry), highlighting the positive impact of gymnastics on skeletal health [10,11,12,13].

Therefore, children and adolescents engaged in gymnastics generally develop higher bone density compared with peers involved in low-impact sports or those who remain inactive [10,11,13,14,15,16]. These benefits extend into adulthood, with former gymnasts maintaining higher bone mineral content than physically inactive individuals [14,17,18,19] and those engaged in other sports [20,21,22,23,24].

However, research on BMD retention in middle-aged and older populations, when the risks of osteopenia and osteoporosis are heightened, remains scarce. Most studies on former gymnasts have focused on younger populations, particularly at elite levels. Bass et al. [20] found that retired elite gymnasts (~25 years old) had significantly higher BMD than matched controls, with no decline years after retiring. Similarly, Scerpella et al. [19,25] observed increased forearm BMD in gymnasts both before and after menarche, with benefits persisting up to four years post-training. Zanker et al. [14] reported 6–10% greater BMD in former gymnasts aged 20–32, while Eser et al. [18], documented greater bone mass and geometry in young females ~6 years post-retirement. Long-term bone benefits were further confirmed by Erlandson et al. [21,22] in individuals who trained between ages 8 and 15.

Only a few studies have examined BMD in retired gymnasts ≥ 45 years. Kirchner et al. [26] studied premenopausal females (29–45 years) and found higher BMD in former gymnasts compared to controls, even after adjusting for other physical activity. Pollock et al. [17] evaluated former female collegiate gymnasts approaching menopause (45 ± 3 years) and reported that, 24 years post-retirement, former gymnasts exhibited higher BMD than controls, although the relative decline was similar in both groups. These findings suggest that early gymnastics practice can retain bone mineralization into early adulthood and potentially mitigate osteoporosis risk. However, similar studies are lacking in individuals over 45 despite the accelerated osteopenia/osteoporosis and increased fracture risk in this age group.

While existing evidence consistently supports the long-term skeletal benefits of early gymnastics participation, the paucity of research involving older adults represents a critical gap, particularly given their growing clinical relevance. Thus, further investigation is needed to determine whether former gymnasts, particularly postmenopausal females, maintain sufficient BMD to lower the risk of osteoporosis. Supporting this link would strengthen the hypothesis that higher peak BMD achieved through youth gymnastics may offer protection against osteopenia in later life.

Addressing this gap requires methodological approaches that balance internal validity—through direct comparisons with matched controls—and external validity by situating findings within broader population norms. Combining individually matched control groups with large population-based reference databases allows for robust between-group comparisons while reducing the risk of conclusions based on small or highly selected samples. Although longitudinal designs would offer stronger causal inference, they demand extended follow-up periods and substantial resources, especially in specific subgroups such as former athletes.

To address these limitations, the present study compared bone mineralization and the prevalence of osteopenia and osteoporosis in individuals aged 45 years and older with a history of competitive artistic gymnastics to those of a matched control group. Physical activity during the previous 10 years was also considered. In addition, former gymnasts’ BMD values were compared with normative data derived from large population-based cohorts. The study was designed as a hypothesis-driven investigation, with predefined outcomes. We hypothesized that former gymnasts would exhibit higher BMD and a lower prevalence of osteopenia and osteoporosis than age-matched non-gymnasts.

## 2. Materials and Methods

### 2.1. Study Population

The group of former gymnasts consisted of individuals aged 45 or older who had competed in artistic gymnastics between 1960 and 1990 in Brazil or Portugal. Recruitment was conducted through the Brazilian Gymnastics Confederation, the Portuguese Gymnastics Federation, and the Gymnastics Federation of the State of Rio de Janeiro, as well as directly contacting former athletes, coaches, and training centers in Brazil and Portugal.

A control group, matched by age and body mass, was recruited from participants in university extension projects in Brazil and Portugal, through outreach via social media. For comparing BMD in former gymnasts with population values, data from the FIBRA Network (Frailty in Brazilian Older Adults, Brazil) and the Research Center for Physical Activity, Health, and Leisure—CIAFEL (Faculty of Sport, University of Porto, Portugal) were used. The FIBRA Network is a multicenter, multidisciplinary longitudinal study focused on investigating the prevalence, characteristics, and risk factors of frailty in the Brazilian population. The CIAFEL conducts prominent research on physical activity for older adults, including regular bone densitometry assessments.

Exclusion criteria included: (a) chronic conditions known to significantly affect bone mineralization (e.g., Cushing’s syndrome, rheumatoid arthritis, systemic lupus erythematosus, skeletal malignancies, or Crohn’s disease); (b) history of chemotherapy or radiotherapy; (c) current pregnancy; (d) Class III obesity (body mass index—BMI ≥ 40 kg/m^2^), and (e) diagnosis of anorexia nervosa or bulimia nervosa. In addition, participants were screened for medical conditions known to markedly affect bone metabolism, including those typically requiring chronic systemic corticosteroid therapy; no participant reported long-term glucocorticoid use, and current breastfeeding was not applicable given the age range of the female sample (≥45 years).

All participants provided written informed consent. The study was approved by the Research Ethics Committee of the University of Rio de Janeiro State (CAAE: 67021322.6.0000.5259).

### 2.2. Study Design

Data were collected during a single visit to the research centers between June 2023 and April 2025. Initially, participants were screened for eligibility by inquiring about their medical history, menopause status, eating disorders, and medication use. Each visit lasted approximately 40 min. Participants first completed a questionnaire assessing physical activity during youth and in the past 10 years. Former gymnasts also reported competitive level, age of initiation, and training volume. Body weight and height were measured, and participants underwent a bone densitometry exam (full body and non-dominant femur). DXA reports were provided to participants and sent electronically.

### 2.3. Procedures

#### 2.3.1. Bone Densitometry

Body composition was assessed using dual-energy X-ray absorptiometry (DXA) at the Laboratory of Physical Activity and Health Promotion (LABSAU, Brazil; Lunar Prodigy Advance™, GE Healthcare, Madison, WI, USA) and at CIAFEL (Portugal; Hologic™ Explorer QDR, Hologic Inc., Marlborough, MA, USA). DXA-derived bone mineral density (BMD) is considered the gold standard for osteoporosis diagnosis and overall bone health assessment due to its high reproducibility, low radiation exposure, and validated predictive value in large cohorts [2,3,27,28]. 

Whole-body scans were conducted by certified professionals with participants in the supine position. Bone mineral content (BMC, g) and areal BMD (g/cm^2^) were determined for the whole body and the non-dominant proximal femur, including the femoral neck and total hip. All procedures adhered to the official guidelines of the International Society for Clinical Densitometry (ISCD) [28].

Scans were scheduled according to individual availability. Participants were instructed to arrive 30 min prior to the scheduled appointment, and each scan lasted approximately 30 min. For the whole-body scan, participants remained supine, relaxed, with legs extended and arms positioned alongside the body. For proximal femur assessment, the non-dominant hip was scanned with the participant in the supine position, the leg extended and internally rotated approximately 15–25° using a positioning device, allowing acquisition of femoral neck and total hip measurements in accordance with the ISCD Official Positions [28]. 

Before each scan, the device was calibrated using a phantom model. Precision and least significant change were determined, with minimum acceptable values set at 2.5% and 6.9%, respectively, for femoral neck BMD. Precision for DXA-derived body composition variables had been previously established for the technicians involved, with coefficients of variation for fat and lean mass measurements typically within the 1–3% range under standardized scanning conditions, although precision was not reassessed specifically for the present study.

For participants with surgical implants (e.g., metal prostheses or surgical staples), half-body scans were performed for whole-body assessment, and values were doubled accordingly; this procedure was required in only one case. The number of individuals present in the examination room was limited to the evaluator and participant at LABSAU and to the participant only at CIAFEL. In both facilities, room temperature was maintained between 21 and 24 °C and relative humidity between 60 and 70%. Participants were instructed to wear light clothing, remove all metal objects, and avoid alcohol and calcium-containing medications for 24 h prior to the examination; fasting was not required.

Normative databases from the National Health and Nutrition Examination Survey (NHANES) 1999–2004, adjusted for White, Black, and Hispanic females and males, were used as reference standards for BMC/BMD values and Z-score calculation [29]. Because it provides a stable young adult reference aligned with World Health Organization (WHO) criteria, the NHANES III database was used to calculate T-scores for whole-body and hip measurements in individuals of both sexes [2,30]. Based on these standards, osteoporosis was defined as a femoral neck T-score ≤ −2.5. Although the ISCD recommends the use of the terms “low bone mass” or “low bone mineral density” when reporting DXA outcomes [28], the term *osteopenia* was used in the present study to denote T-scores between −1.0 and −2.5, in accordance with WHO criteria and to facilitate comparison with previous epidemiological studies.

#### 2.3.2. Anthropometric Measurements

Height and body weight were measured by a trained researcher, with participants wearing light clothing and no shoes. Height was measured to the nearest 0.1 cm using wall-mounted stadiometers. Body weight was measured to the nearest 0.1 kg using an electronic scale—LABSAU (Filizola^TM^, São Paulo, SP, Brazil) and CIAFEL (SECATM 899, Hamburg, Germany). The BMI was calculated (kg/m^2^).

#### 2.3.3. Appendicular Muscle Mass Index—Muscle Mass Classification

Appendicular muscle mass was calculated as the lean mass of arms and legs (kg) and was used to calculate the Appendicular Skeletal Muscle Mass (ASM) index [ASM (kg)/height (m^2^)] [31]. Cut-off points of ASM index were used to identify potential sarcopenia (5.45 Kg/m^2^ for females and 7.26 Kg/m^2^ for males) [31]. Those cut-off values were defined as two standard deviations below sex-specific means of reference data obtained in the Rosetta Study with adults aged 18–40 years [32].

#### 2.3.4. Physical Activity Inventory

Physical activity during youth (10–20 years) (PA-Youth) and in the past 10 years (PA-10) was estimated using questions adapted from a previous study on former gymnasts aged 35–40 years [26]. Participants reported the frequency (sessions/week), duration (minutes/session), and perceived intensity (ordinal scale: 1 = light to 4 = very hard) of physical activity during youth (10–20 years) and over the previous 10 years. Training volume was estimated using the number of years, months per year, days per week, and hours per day practiced in each period, allowing calculation of total accumulated hours and average weekly exposure. There was no restriction of exercise/sport participation in both former gymnasts of control groups. Former gymnasts additionally reported the duration of their competitive career and associated training volume.

Intensity ratings were used exclusively to weigh activity volume and estimate total exposure, not to classify or compare specific activity modalities (e.g., high- vs. low-impact or weight-supported activities). This decision was based on the limited variability in reported intensity within groups—similar among controls and consistently high among former gymnasts—and the recognized imprecision of retrospective, impact-specific activity estimates over extended periods. Accordingly, PA-10 was treated as a global indicator of sustained mechanical loading in adulthood and used as an adjustment variable in the analyses.

#### 2.3.5. Statistical Analysis

Normality was tested for each outcome using the full dataset within each group, applying the Shapiro–Wilk test and supported by standard normal probability plots. The W statistic rejected the normality assumption for some variables (T-scores, Z-scores, whole-body BMC), but never across all groups. In these cases, the plot of z-values (calculated from ordered deviations from the mean, i.e., residuals) against expected values from a Gaussian distribution showed a general lack of fit, with the data forming a distinct S-shaped pattern. Nevertheless, the normality assumption was considered sufficiently met to justify the use of parametric tests.

The present study was hypothesis-driven rather than exploratory. All primary comparisons and covariate adjustments were specified a priori based on existing evidence indicating long-term skeletal benefits of early-life participation in high-impact sports. Therefore, analyses were not adjusted for multiple testing, as they were conducted to test predefined hypotheses rather than to explore associations post hoc.

Differences between Brazilian and Portuguese former gymnasts were examined using independent-sample Student’s *t* tests, while categorical variables were compared using chi-square tests (χ^2^). Comparisons between former gymnasts and control participants were also performed by sex using *t* tests for all continuous variables. In addition, differences in bone mineralization outcomes were adjusted for age and physical activity over the last 10 years (PA-10) using analysis of covariance (ANCOVA). 

Stratified comparisons between former gymnasts and controls were also conducted by age group (45–59 years and ≥60 years). Comparisons with the Brazilian and Portuguese reference populations were carried out using the independent samples *t*-test, for both the total sample and age-stratified groups. Statistical significance was set at *p* ≤ 0.05 for all analyses, which were conducted using SPSS version 20 (IBM, Chicago, IL, USA).

Post hoc statistical power for the primary outcomes (bone densitometry variables) was assessed using G*Power version 3.1.5 (University of Düsseldorf, Düsseldorf, Germany) based on comparisons with control groups and reference populations. 

Effect sizes were calculated as Cohen’s d (using pooled standard deviations from independent *t*-tests) and partial eta squared (ηp^2^) for ANCOVA analyses. Statistical power was then estimated assuming α = 0.05 and the respective sample sizes.

## 3. Results

### 3.1. General Characteristics of the Gymnasts in Brazil and Portugal

A total of 65 former gymnasts participated in the study, 45 from Brazil and 20 from Portugal, aged 45–84 years (32 males; 59 White, 3 Asian, 3 Black). Of these, 41 competed internationally and 24 at national/regional levels. Overall, participants from both countries showed equivalent profiles in demographic, clinical, and physical activity variables (Supplementary Material, Table S1). Whole-body BMC, BMD, femoral BMD (neck and total), and related Z- and T-scores were also statistically equivalent and generally above age norms. No osteoporosis was found. Osteopenia (femoral neck) occurred more in females than males in Brazil (seven females, one male) and Portugal (four females, zero male). Appendicular skeletal muscle mass index was comparable, with only two Brazilian males classified with low muscle mass that could be compatible with sarcopenia. Data on gymnastics training (age of beginning, duration, volume) were also consistent across countries, supporting their inclusion as a single group for further comparisons.

### 3.2. Comparison Between Gymnasts and Controls

Table 1 summarizes data for 65 former gymnasts and 91 control participants (37 males; 68 White, 23 Black, age 45–87 years). Both groups were similar in age, height, body mass, and BMI, generally within normal or overweight ranges. Among females, 26 former gymnasts (4 months–27 years of onset) and 50 controls (6 months–37 years of onset) were postmenopausal, with comparable age at menopause and wide variability in time since onset. Nine former gymnasts (1–16 years) and seven controls (1–18 years) reported hormone or calcium therapy.

A full description of PA-10 and PA-Youth by sex is provided in the Supplementary Material (Table S2). As expected, PA-Youth differed significantly between groups, while PA-10 showed no significant differences. For PA-10, 10 former gymnasts (2 males, 8 females) and 10 controls (5 males, 5 females) reported inactivity. Among active participants, common activities of former gymnasts were strength training, walking/running, and dance for females; and walking/running, strength training, and cycling for males. In the control group, among other activities, control females reported strength training, walking/running, and aquatic exercise; males reported strength training, running/walking, and cycling. Activity profiles did not differ significantly between groups. 

During youth, most former gymnasts (n = 56) practiced only artistic gymnastics; international-level gymnasts reported no other physical activity, while regional/national athletes also engaged in dance, tumbling, and swimming, among others. In this group, PA-Youth was higher than PA-10, with gymnastics being systematically classified as high intensity, frequency >5 sessions/week, 5–8 h/week. Among controls, PA-Youth was more variable and significantly lower than in former gymnasts (*p* < 0.001), reflecting unstructured, low-frequency leisure activity (≈2.5 days/week). No PA-Youth was reported by 24 female controls; others practiced ballet/dance, swimming, or volleyball. All but three control males reported some activity, mostly soccer, walking/running, and calisthenics. Since a substantial number of control female participants reported no PA-Youth (almost half of 54), we performed additional comparisons between former gymnasts vs. those who were inactive and moderately active, and no additional difference was found (*p* > 0.42).

Chronic conditions were reported by 27 former gymnasts (11 females, 16 males) and 55 controls (33 females, 22 males). Cardiovascular issues, musculoskeletal pain, and thyroid disorders were most common. The prevalence of chronic diseases was similar across groups, except for hypertension among females. Overweight/obesity rates were comparable among males, but significantly higher among control females. Former gymnasts had lower fat mass and body fat percentage (*p* < 0.05), though lean and muscle mass did not differ. Obesity was more common in control females (10 vs. 4, *p* < 0.05), while the difference among males was not significant. DXA-confirmed fat percentages ranged from 45.6 to 55.8% in obese females and from 30.4 to 41.9% in males. Total and relative fat mass were higher in controls, but ASM and lean mass were similar. Low muscle mass was more prevalent in controls (11 cases: 5 females or 9.3% and 6 males or 16.2%, 50–74 years) than in gymnasts (2 males or 3.1%, 66 and 84 years). 

Bone densitometry comparisons revealed distinct patterns by sex and anatomical site. Significant differences were limited to the femoral neck T-score in females and to the Z-scores at the femoral neck and total femur in both sexes. Female gymnasts showed T-scores nearly twice as high as controls, and both male and female gymnasts exhibited Z-scores 5–10 times greater than those of their respective control groups. Effect sizes and corresponding statistical power for bone densitometry are available in the Supplementary Material (Table S3). Among males, the femoral neck Z-score showed a moderate effect size (d = 0.56; power = 0.67), and the total femur Z-score a similar magnitude (d = 0.56; power = 0.68). In females, the femoral neck T-score demonstrated a moderate-to-large effect (d = 0.68; power = 0.94), and the Z-score at the same site an even stronger difference (d = 0.79; power = 0.99). The total femur Z-score also showed a moderate effect (d = 0.46; power = 0.54). All other comparisons, including whole-body and femoral BMD, BMC, and T-scores, showed small effect sizes (|d| ≤ 0.33) and low statistical power (<0.37), indicating no meaningful differences between groups. 

Overall, these findings indicate that former gymnastics participation confers lasting, site-specific skeletal benefits, particularly in weight-bearing regions of the femur. No cases of osteoporosis were identified among former gymnasts, whereas two females (60 and 67 years) and one man (65 years) in the control group met the diagnostic criteria. Osteopenia assessed at the femoral neck was more prevalent in controls (26 females and 5 males; 51–77 years) than in former gymnasts (11 females and 1 man; 50–76 years). When osteopenia and osteoporosis diagnoses were combined, former gymnasts showed a clear advantage over controls in the relative prevalence of these conditions—males: 3.1% vs. 16.2% (*p* = 0.01), respectively; females: 36.4% vs. 51.8% (*p* = 0.045), respectively.

ANCOVA results (Table 2), adjusted for age and PA-10, confirmed higher femoral T- and Z-scores in former gymnasts. PA-10 did not show a significant independent effect on any of the comparisons. Although some adjustments were observed for age, these did not alter the differences between former gymnasts and controls. Among males, partial eta squared values ranged from negligible to moderate (ηp^2^ = 0.000–0.126), with corresponding statistical power between 0.05 and 0.72. The largest effects were observed for the total femur Z-score (ηp^2^ = 0.126; power = 0.72) and femoral neck Z-score (ηp^2^ = 0.102; power = 0.67). In females, ηp^2^ values were generally small (0.000–0.090) with power up to 0.55, indicating modest group differences. The most notable effects in females were found for the femoral neck T-score (ηp^2^ = 0.078; power = 0.48), the femoral neck Z-score (ηp^2^ = 0.084; power = 0.53), and the total femur Z-score (ηp^2^ = 0.090; power = 0.55), all indicating a trend toward higher bone mineral density in former gymnasts (Supplemental Material, Table S4). These results suggest limited but consistent evidence of higher femoral bone density indices among former gymnasts, implying lasting skeletal adaptations despite adjustment for age and physical activity.

Given the isolated effect of age in several comparisons, an additional stratified analysis (Table 3) was performed for participants aged 45–59 and ≥60 years. Although bone mineralization advantages were most pronounced in the younger group, they persisted in older participants.

As for hormonal therapy, there was no case among males. In females, to address the potential impact of hormone or calcium replacement therapy on bone mineralization, BMD and related indices were compared between postmenopausal former gymnasts and controls in additional analyses stratified by replacement therapy status (Supplement Material, Table S5). No consistent association between replacement therapy and bone outcomes was identified in either group. Former gymnasts receiving therapy showed marginally higher whole-body Z-scores, but this was not observed at other skeletal sites or among controls. Femoral neck Z-scores were consistently higher in gymnasts than in controls, irrespective of therapy status. Among non-users, gymnasts were younger and had a shorter time since menopause, which may have contributed to differences. Overall, replacement therapy appeared to exert a limited influence on BMD compared with prior gymnastics participation.

### 3.3. Comparison with Reference Populations

Table 4 summarizes femoral BMD data comparing former gymnasts with two reference populations: a Brazilian cohort (n = 828; 354 males; mixed ethnic backgrounds; age range 45–96 years) and a Portuguese cohort (n = 1089; 528 males; all White; age range 45–88 years). Former female gymnasts were younger and had lower body mass and BMI than both reference groups. Among males, ages were comparable across groups, but former gymnasts exhibited lower body mass and BMI than their Portuguese counterparts. Overweight and obesity were more prevalent in the reference populations, particularly in females. Significant differences in males were observed only in comparison with the Portuguese data.

Bone densitometry analyses showed moderate to very large effect sizes in most comparisons, associated with statistical power values approaching 1.0, indicating robust differences between former gymnasts and the reference populations. A complete summary of effect sizes and statistical power is provided in the Supplementary Material (Table S6). 

Former gymnasts consistently exhibited higher whole-body BMC and BMD, with markedly elevated T- and Z-scores (up to fourfold greater than those of the reference populations), a difference that remains noteworthy even after accounting for the younger age of female gymnasts. In males, these differences were evident across all bone variables (d = 1.01–1.44 for whole-body and d = 0.74–1.46 for femoral sites, power > 0.98), confirming substantially greater bone density and preserved mass in load-bearing regions. The advantage was most pronounced relative to the Portuguese cohort (d ≈ 0.65–1.46, power = 0.95–1.00), whereas differences compared with the Brazilian population were moderate (d ≈ 0.22–0.86, power = 0.45–0.99). Among females, the effects were smaller but remained meaningful: former gymnasts exhibited higher whole-body and femoral neck mineralization compared with the Portuguese population (d = 0.65–1.89, power > 0.95), while their values were largely comparable to those of Brazilian counterparts (d < 0.55, power < 0.85).

In general, the large effect sizes and high statistical power support the conclusion that a background in gymnastics related to greater bone mass and density, particularly among males and relative to the Portuguese cohort. In consequence, osteopenia was notably less frequent among former gymnasts—especially males—and no cases of osteoporosis were observed, contrasting with the 6–12% prevalence reported in the reference populations. These findings highlight the long-term osteogenic benefits of impact-based training for preserving skeletal health with advancing age.

To account for age effects, a stratified analysis was conducted for participants <60 and ≥60 years (Table 5). In the <60 group, anthropometric characteristics were similar, though female gymnasts had significantly lower overweight/obesity prevalence. Both male and female gymnasts under 60 showed higher BMD, T-, and Z-scores than Brazilian and Portuguese peers. Osteopenia was notably less frequent in male gymnasts and moderately lower among female gymnasts. No osteoporosis was observed in any gymnast, while reference population rates ranged from 2 to 8%.

Among participants aged ≥ 60 years, Brazilian controls were older, and Portuguese males had slightly higher obesity prevalence than gymnasts. Former gymnasts maintained higher whole-body BMD and BMC. Male gymnasts continued to show superior femoral Z-scores (4–6 times higher), while advantages in females were smaller, particularly versus Portuguese controls. Osteopenia remained substantially lower in male gymnasts; among females, prevalence was moderate and statistically similar (57% vs. 45–52%). No gymnast showed osteoporosis, contrasting with 9–13% prevalence in the reference groups.

## 4. Discussion

This study demonstrates that adults aged ≥ 45 years with a history of competitive artistic gymnastics exhibit superior bone health compared with age-matched non-athletes and population-based references. Former gymnasts had higher femoral BMD, favorable T- and Z-scores, and lower prevalence of osteopenia and osteoporosis, supporting the concept of a “bone bank” established during youth through high-impact, osteogenic activities.

The focus on BMD and related T- and Z-scores must be clarified. Although bone microarchitecture variables such as cortical thickness and trabecular density can provide mechanistic insight into skeletal adaptations, their assessment was not pursued in this study. Standard DXA is a two-dimensional technique and does not directly measure bone microarchitecture; DXA-derived structural indices are indirect, device-dependent, and insufficiently standardized across manufacturers, limiting their reliability, particularly in multicenter studies. In older adults, age-related remodeling and degenerative changes further compromise the interpretability of these measures. Importantly, BMC, BMD, and their corresponding T- and Z-scores at clinically relevant sites remain the gold standard for osteoporosis diagnosis and fracture risk prediction. Therefore, focusing on these outcomes provides the most reliable, comparable, and clinically meaningful evaluation of bone health in the present cohort.

It is worth noticing that in multicenter studies employing DXA systems from different manufacturers, absolute areal BMD and standardized indices (T- and Z-scores) may diverge and should not be interpreted interchangeably. T- and Z-scores are derived from manufacturer-specific, sex- and age-matched reference databases and incorporate both the mean and variability of the reference population, rather than representing direct transformations of observed BMD values. As a result, individuals, or groups with comparable or even slightly lower absolute BMD may exhibit more favorable standardized scores depending on their relative position within the appropriate reference distribution. This phenomenon is particularly relevant at the femoral neck, where inter-manufacturer differences in calibration and reference data are well documented. Accordingly, and in line with the ISCD Official Positions [28], interpretation in the present study prioritized standardized scores and diagnostic classifications to minimize device-related bias and enable valid comparisons across populations.

Gymnastics produces ground reaction forces up to 10–15 times body weight, offering potent mechanical stimuli that promote bone accrual during growth [2,6,7,16]. These high-impact forces drive skeletal adaptations such as increased cortical thickness, enhanced trabecular density, and favorable geometric changes [8,12], all of which contribute to greater fracture resistance, a key factor in maintaining skeletal health across the lifespan [4,15,21,22]. Notably, these structural benefits may persist for decades after training ceases, highlighting the long-term impact of early-life participation in high-impact sports. Evidence suggests that even after the discontinuation of training and competition, a portion of these skeletal adaptations is retained [8].

Similarly, high-impact sports involving jumping, landing, and weight-bearing activities induce lasting structural changes in bone [12,13,23,24]. Early participation in such activities has been shown to enhance peak bone mass and produce residual improvements in bone mineralization and architecture that may persist well into adulthood [4,13,14,15,18,19,20,21,22,25].

Sex-specific patterns were evident: males retained skeletal benefits more steadily, while this lasting effects in females ≥ 60 years were inconsistent, likely due to postmenopausal estrogen decline. Although whole-body BMD did not differ significantly, former female gymnasts consistently displayed higher femoral T- and Z-scores than their age-matched controls, highlighting clinically relevant protection during accelerated bone loss after menopause [1,27]. Early high-impact training appears to partially offset hormonal declines, contributing to a lower prevalence of osteopenia in females. In males, advantages were more consistent across ages, reflecting the absence of abrupt hormonal changes [6,33]. 

Bone mineralization reflects the cumulative effects of mechanical loading over time rather than short-term fluctuations in physical activity [4,6]. Because bone remodeling and age-related bone loss occur gradually, physical activity sustained over the previous decade provides a more biologically meaningful indicator of habitual skeletal loading than current activity levels. Long-term measures better capture consistent exposure to mechanical stimuli, reduce the influence of temporary behavioral changes, and are particularly relevant in middle-aged and older adults, when bone loss accelerates. Consequently, physical activity during the last 10 years shows a stronger and more reliable association with BMD than contemporaneous activity alone, supporting its inclusion as a key covariate in the present analyses. 

PA-10 levels were similar between groups, whereas former gymnasts reported higher PA-Youth, reinforcing adolescence as a critical window for maximizing peak bone mass [6,34]. Longitudinal studies suggest that up to 50% of adult BMD derives from peak values attained in youth [4,35], particularly at fracture-prone sites such as the hip, femoral neck, and lumbar spine [33,35]. ANCOVA confirmed that skeletal benefits persisted after adjusting for age and PA-10, emphasizing the cumulative advantage model, whereby higher peak bone mass provides a long-term skeletal resilience [4,33,36].

The starting age of gymnastics training could be a concern due to the argument that late menarche or oligomenorrhea commonly observed in girls exposed to high training volumes may impair skeletal development. Unfortunately, we were unable to directly address this issue in the present study. However, this premise has been questioned. For instance, Lindholm et al. [37] reassessed 22 elite Swedish gymnasts (19-23 years) who had been followed longitudinally from adolescence to early adulthood (11-14 to 16-19 years). Despite late menarche, delayed pubertal development, and irregular menstrual cycles, no differences in skeletal development were observed when compared with age-matched controls whose menarche occurred earlier. These findings suggest that, although bone mineralization may be delayed, the mechanical loading associated with artistic gymnastics may compensate for this delay and help preserve bone health. Moreover, as shown in Table S1, the age at which gymnastics training began and the duration of training were very similar between Brazilian and Portuguese participants, with relatively low variability. Therefore, any potential influence of training onset age is likely to have been evenly distributed across the sample.

Hormone and calcium therapy, known to influence bone mineralization in postmenopausal females [38,39], had minimal influence on BMD in our sample due to their low prevalence. No consistent BMD differences were observed between users and non-users. Whole-body Z-scores were higher in gymnasts on therapy, but this was not replicated at other sites or in controls. Femoral neck Z-scores remained higher in gymnasts regardless of therapy, suggesting that athletic history exerted a stronger effect. Non-users among gymnasts were younger with shorter menopause duration, which may have conferred additional protection given the steep early postmenopausal decline [5,27]. These findings confirm that impact exercise during youth yield lasting benefits, comparable to hormone therapy. A recent review [40] concluded that both interventions help preserve BMD, though combined therapy is most effective. Given safety concerns with hormone therapy, exercise remains a cornerstone of osteoporosis prevention.

Comparisons with large population-based cohorts reinforced these results: Former gymnasts exhibited higher whole-body and femoral BMD, more favorable T- and Z- scores, and lower prevalence of osteopenia and osteoporosis than Brazilian and Portuguese references. Notably, no gymnast was diagnosed with osteoporosis, compared with 6–12% prevalence in the general population. Benefits were most evident before age 60; in females ≥ 60 years, these effects were less consistent probably due to prolonged postmenopausal bone loss, whereas males retained advantages across all ages. These sex-specific trajectories highlight interactions between early skeletal loading and hormonal influences on aging, consistent with functional reserve and cumulative advantage concepts [4,5,36,41].

Previous studies demonstrated that gymnasts retained skeletal benefits into midlife [17,26]. The present study extends this evidence to individuals ≥ 60 years, showing advantages even after decades of withdrawal from sport. These adaptations likely explain the absence of osteoporosis and the reduced prevalence of osteopenia in our sample [4,11]. In participants under 60 years, former gymnasts consistently displayed higher BMD, positive T- and Z-scores, and no osteoporosis, supporting the concept of a “bone bank” accrued during youth [22,34,36,42]. 

Among participants ≥ 60 years, male gymnasts maintained clear BMD advantages and low osteopenia prevalence, reflecting a lower susceptibility to bone loss [43]. In females, those benefits appeared reduced: osteopenia prevalence reached 57%, aligning with rates observed in reference populations, while osteoporosis remained undetected. Although the small sample of older females limits interpretation, these findings provide novel evidence of skeletal preservation associated with early gymnastics. The absence of osteoporosis in both sexes, contrasting with 9–13% prevalence in Brazilian and Portuguese cohorts, further support the hypothesis that high mechanical bone loading in youth protects against severe demineralization later in life [3,4,6,9,24,34,36].

Sex differences were evident, with males retaining youth-acquired skeletal benefits more consistently, whereas reproductive aging constrained long-term protection in females. Similar patterns have been observed in other high-impact cohorts [42,44]. The abrupt estrogen decline at menopause accelerates bone loss, explaining why early training cannot fully offset postmenopausal deficits [5,41,43]. Thus, mechanical loading during growth enhances peak bone mass, but these long-term effects seem to be modulated by hormonal changes across the lifespan of females.

We acknowledge that the perimenopausal transition is associated with accelerated bone loss and may increase variability in bone outcomes among females who are recently postmenopausal. In the present study, group comparisons were adjusted for chronological age rather than menopausal stage, as detailed staging data were not available for all participants; this represents a limitation and may have contributed to residual variability in female bone measures. Nevertheless, the age range examined reflects the period in which long-term skeletal consequences of earlier-life mechanical loading become clinically relevant. Outcomes were interpreted using standardized T- and Z-scores, which account for age-related bone changes, and the consistent differences observed between former gymnasts and controls are unlikely to be explained solely by menopausal transition effects.

Taken together, the findings suggest that early participation in competitive artistic gymnastics is associated with more favorable bone mineral density and body composition in later life. Benefits appear more robust in males, while reproductive aging, particularly postmenopausal estrogen decline, partially offsets these benefits in females. Lifelong physical activity remains important for preserving musculoskeletal health, but structural advantages gained through high-impact exercise during youth seem to play a foundational role. Noteworthy, exercise that loads bone also improves qualities of the muscle, an effect that osteoporosis drugs do not provide.

This study has strengths and limitations. Strengths include the use of standardized DXA assessments, comparisons with large population databases, and a relatively large sample compared to previous studies on former gymnasts. However, limitations should be acknowledged. The cross-sectional design precludes causal inference, and the small subgroup sizes, especially among females aged 60 and over, limit statistical power and increase susceptibility to individual variability. Cross-calibration between DXA scanners across sites was not possible, as the same individuals could not be assessed in both countries. Physical activity was assessed retrospectively, and key factors such as hormonal status, medication use, dietary intake, and lifestyle behaviors were not systematically controlled. Sex hormone therapy data in the population databases were not available. Finally, direct comparisons with reference populations must be interpreted with caution, as the datasets were obtained in different methodological and temporal contexts and are influenced by cultural, nutritional, and socioeconomic differences beyond the scope of this study’s control.

## 5. Conclusions

Former gymnasts aged ≥ 45 years demonstrated a more favorable bone health profile than age-matched controls and large reference populations from Brazil and Portugal, characterized by higher femoral and whole-body BMD, more favorable standardized scores, and a lower prevalence of osteopenia, with no cases of osteoporosis. These advantages were most evident in females aged 45–60 years, whereas skeletal protection in females aged ≥ 60 years was less consistent, likely reflecting the cumulative effects of prolonged postmenopausal bone loss. In contrast, males retained bone health advantages across all age groups, suggesting the establishment of durable skeletal reserves through early-life training.

Importantly, these findings should not be interpreted as indicating that youth physical activity must reach the high training volumes typical of artistic gymnastics to confer skeletal benefits. Rather, they highlight the long-term osteogenic potential of high-impact, multidirectional loading during growth. Although former elite gymnasts represent a highly trained and selected group, the underlying biological principle—that appropriately applied mechanical loading during youth enhances peak bone mass with lasting consequences—is broadly applicable. Thus, while most youth do not require elite-level training, regular participation in weight-bearing and impact-based activities remains a realistic and evidence-based strategy to support lifelong bone health. Nevertheless, maintenance of bone health in later life remains essential, particularly for postmenopausal females, underscoring the need for continued physical activity and targeted preventive strategies. Future longitudinal and multicenter studies incorporating hormonal, nutritional, and behavioral factors are warranted to better define the magnitude and persistence of these early-life skeletal adaptations, and whether they can be enhanced through continued physical activity or targeted interventions.

## 6. Implications for Health Promotion

Our findings indicate that early engagement in high-impact, weight-bearing exercise is associated with sustained skeletal benefits, supporting the concept that mechanical loading during youth contributes to long-term bone health. Participation in activities such as artistic gymnastics, jumping, or other multidirectional sports aligns with recommendations from organizations including the World Health Organization, the American College of Sports Medicine, and the International Osteoporosis Foundation, which emphasize vigorous, osteogenic exercise to enhance peak bone mass and lower fracture risk later in life.

The prepubertal and pubertal periods appear to be critical windows for bone accrual; accordingly, structured high-impact exercise performed three to four times per week under supervised and safe conditions may be beneficial. In adulthood, particularly following menopause, combined exercise programs incorporating moderate-impact, resistance, and balance training have been shown to help maintain bone mass and attenuate postmenopausal bone loss. Overall, these findings support a lifespan approach to skeletal health, in which early mechanical loading contributes to the establishment of a robust skeletal framework that, when maintained through continued physical activity, may reduce the risk of osteopenia and osteoporosis in later life.

## Figures and Tables

**Table 1:** Characteristics of former gymnasts and control group—mean (standard deviation).

Variable	Males		*Females*	
	Former Gymnasts (n = 32)	Controls (n = 37)	*Former Gymnasts* *(n = 33)*	*Controls* *(n = 54)*
**General**				
Age (year)	60 (9)	61 (8)	*55 (8)*	*60 (9)*
Height (m)	1.69 (0.05)	1.72 (0.07)	*1.58 (0.05)*	*1.59 (0.01)*
Body mass (kg)	78.1 (11.5)	82.1 (11.6)	*61.1 (9.2)*	*67.9 (11.5)*
BMI (kg/m^2^)	27.3 (3.9)	27.6 (2.9)	*24.6 (3.6)*	*26.9 (4.5)*
Menopause onset (year)	-	-	*49 (2)*	*48 (5)*
Years after menopause onset (year)	-	-	*7.4 (8.2)*	*12.3 (9.1)*
PA-Youth (points)	10,836 (3912) *	**1183 (1084)**	11,632 (5790) *	***666 (1092)***
PA-10 (points)	1.045 (1.170)	880 (936)	*734 (891)*	*543 (495)*
**Artistics Gymnastics (AG)**				
Age of AG beginning (year)	10 (3)	-	*8 (3)*	*-*
Time of AG training (year)	15 (5)	-	*11 (4)*	*-*
Training frequency (day/week)	6 (1)	-	*6 (1)*	*-*
Training volume (h/day)	5 (1)	-	*5 (1)*	*-*
**Chronic Disease**				
Diabetes (n) (%)	2 (6)	5 (13)	*-*	*9 (17)*
Hypertension (n) (%)	9 (28)	14 (38)	*1 (3)*	***19 (35) ^a^***
Hypo/Hyperthyroidism (n) (%)	1 (3)	-	*4 (12)*	*1 (2)*
Dyslipidemia (n) (%)	1 (3)	1 (3)	*3 (9)*	*1 (2)*
Cervical/Lumbar pain (n) (%)	2 (6)	-	*-*	*-*
Chronic pain (n) (%)	-	-	*1 (3)*	*1 (2)*
Cardiac disease (n) (%)	1 (3)	1 (3)	*-*	
Respiratory disease (n) (%)	-	1 (3)	*2 (6)*	*1 (2)*
Liver disease (n) (%)	-	-	*-*	*1 (2)*
Overweight/obesity (n) (%)	22 (69)	29 (78)	***11 (33)***	***34 (63) ^a^***
**Bone densitometry**				
BMC whole-body (g)	2920.5 (324.6)	2943.0 (378.1)	*2118.4 (238.7)*	*20,629 (287.5)*
BMD whole-body (g/cm^2^)	1.312 (0.130)	1.302 (0.136)	*1.108 (0.079)*	*1.095 (0.106)*
Z-Score, whole-body	1.28 (0.99)	0.80 (1.24)	*0.84 (0.64)*	*0.75 (0.97)*
BMD femur neck (g/cm^2^)	0.996 (0.106)	1.02 (0.141)	*0.894 (0.150)*	*0.893 (0.119)*
T-Score, femur neck	−0.08 (0.65)	−0.19 (0.95)	−0.51 (0.91) *	***−1.02 (0.46)***
Z-Score, femur neck	0.64 (0.66) ^*^	**0.12 (1.04)**	0.58 (0.94) *	***0.02 (0.60)***
BMD femur total (g/cm^2^)	1.084 (0.112)	1.066 (0.151)	*0.953 (0.140)*	*0.935 (0.114)*
T-Score, femur total	0.56 (0.79)	0.46 (1.19)	*−0.25 (1.04)*	*−0.57 (0.91)*
Z-Score, femur total	0.47 (0.72) ^*^	**−0.04 (1.00)**	0.54 (0.86) ^*^	***0.16 (0.82)***
**Body composition**				
Fat mass (g)	20,484 (8160) ^*^	**25,505 (5112)**	20,436 (7204) ^*^	***28,330 (8272)***
Relative fat (%)	26.4 (7.6) ^*^	**32.0 (4.9)**	33.6 (7.4) ^*^	***41.6 (6.6)***
Lean mass (g)	54,488 (5086)	53,124 (6344)	*38,230 (3923)*	*37,642 (4480)*
ASM/Height^2^ (kg/m^2^)	8.65 (0.97)	8.53 (1.63)	*6.67 (0.68)*	*6.79 (1.12)*

AG: Artistic gymnastics; ASM: appendicular skeletal muscle mass; BMC: bone mineral content; BMD: areal bone mineral density; BMI: body mass index; PA-Youth: physical activity volume in youth; PA-10: physical activity volume in the past 10 years; *: difference between former gymnasts and controls for a given sex by Student’s *t*-test (*p* < 0.05); ^a^: difference between former gymnasts and controls for a given sex by chi-square test (*p* < 0.05). Significant differences are highlighted in bold type.

**Table 2:** Bone mineral density (BMD) results in former gymnast and control groups—mean (95% CI) adjusted by ANCOVA using physical activity volume over the past 10 years (PA-10) and age as covariates ^a^.

Variable	Males		*p*-Value	*Females*		*p*-*Value*
	Former Gymnasts (n = 32)	Controls (n = 37)		*Former Gymnasts* *(n = 33)*	*Controls* *(n = 54)*	
BMD whole-body (g/cm^2^)	1.310 (1.262; 1.358)	1.303 (1.259; 1.348)	0.843	*1.088* *(1.058; 1.118)*	*1.107* *(1.084; 1.130)*	*0.334*
Z-Score, whole-body	1.27 (0.85; 1.70)	0.80 (0.41; 1.20)	0.113	*0.81* *(0.48; 1.13)*	*0.79* *(0.52; 1.03)*	*0.997*
BMD, femur neck (g/cm^2^)	0.996 (0.950; 1.040)	1.012 (0.971; 1.055)	0.556	*0.873* *(0.829; 0.916)*	*0.906* *(0.872; 0.940)*	*0.245*
T-Score, femur neck	−0.08 (−0.39; 0.21)	−0.19 (−0.46; 0.11)	0.681	***−0.63*** ***(−0.95; −0.32)***	***−1.02*** ***(−1.26; −0.77)***	***0.048***
Z-Score, femur neck	**0.64** **(0.32; 0.96)**	**0.17** **(−0.13; 0.47)**	**0.034**	***0.44*** ***(0.14; 0.74)***	***0.04*** ***(−0.19; 0.27)***	***0.044***
BMD femur total (g/cm^2^)	1.084 (1.036; 1.132)	1.066 (1.021; 1.111)	0.595	*0.933* *(0.891; 0.975)*	*0.947* *(0.915; 0.979)*	*0.598*
T-Score, femur total	0.56 (0.18; 0.94)	0.46 (0.11; 0.81)	0.716	*−0.44* *(−0.76; −0.12)*	*−0.48* *(−0.72; −0.23)*	*0.869*
Z-Score, femur total	**0.48** **(0.16; 0.79)**	**−0.05** **(−0.34; 0.25)**	**0.019**	***0.51*** ***(0.18; 0.84)***	***0.18*** ***(−0.08; 0.43)***	***0.022***

BMD: areal bone density; ^a^ Covariates included in the model were evaluated at the following values: PA-10 = 956.38, Age = 60.75 (males), and PA-10 = 615.49, Age = 58.30 (females). In no case PA-10 had a significant independent effect. Significant differences are highlighted in bold type.

**Table 3:** Comparison of bone densitometry variables between former gymnasts and control individuals by age subgroup (<60 years and ≥60 years)—mean (standard deviation).

Age < 60 Years				
Variable	Males		*Females*	
	Former Gymnasts (n = 16)	Controls (n = 14)	*Former Gymnasts* *(n = 26)*	*Controls* *(n = 25)*
Age (year)	54 (5)	53 (5)	*52 (4)*	*52 (3)*
Menopause onset (year)	-	-	*49 (1), n = 19*	*49 (5), n = 21*
Years after menopause onset (year)	-	-	*4 (4), range 1–11*	*4 (5), range 1–17*
BMD whole-body (g/cm^2^)	1.295 (0.142)	1.306 (0.127)	*1.124 (0.075)*	*1.153 (0.084)*
Z-Score, whole-body	0.89 (1.11) *	**0.56 (1.32)**	*0.81 (1.02)*	*1.00 (0.87)*
BMD femur neck (g/cm^2^)	1.004 (0.117)	1.009 (0.118)	*0.912 (0.160)*	*0.951 (0.104)*
T-Score, femur neck	0.08 (0.63)	−0.19 (0.86)	*−0.43 (1.00)*	*−0.62 (0.74)*
Z-Score, femur neck	0.65 (0.57) *	**−0.18 (0.62)**	0.52 (1.03) *	***0.17 (0.68)***
BMD femur total (g/cm^2^)	1.071 (0.122)	1.054 (0.123)	*0.976 (0.147)*	*0.973 (0.097)*
T-Score, femur total	0.45 (0.98)	0.37 (0.97)	−0.07 (1.07) *	***−0.31 (0.77)***
Z-Score, femur total	0.29 (0.34) *	**−0.29 (0.43)**	0.58 (0.40) *	***0.17 (0.76)***
**Age ≥ 60 Years**				
**Variable**	**Males**		***Females***	
	**Former Gymnasts** **(n = 16)**	**Controls** **(n = 23)**	***Former Gymnasts*** ***(n = 7)***	***Controls*** ***(n = 29)***
Age (year)	66 (7)	66 (5)	*68 (6)*	*67 (6)*
Menopause onset (year)	-	-	*49 (4), n = 7*	*47 (4), n = 29*
Years after menopause onset (year)	-	-	*19 (7), range 8–27*	*19 (8), range 4–37*
BMD whole-body (g/cm^2^)	1.329 (0.120)	1.299 (0.145)	*1.047 (0.065)*	*1.045 (0.992)*
Z-Score, whole-body	1.68 (0.56) *	**0.94 (0.30)**	0.96 (0.49) *	***0.54 (0.31)***
BMD femur neck (g/cm^2^)	0.988 (0.097)	1.013 (0.155)	*0.824 (0.084)*	*0.843 (0.112)*
T-Score, femur neck	−0.24 (0.66)	−0.19 (1.10)	*−1.34 (0.44)*	*−1.37 (0.81)*
Z-Score, femur neck	0.65 (0.36) *	**0.35 (0.49)**	0.33 (0.44) *	***−0.12 (0.47)***
BMD femur total (g/cm^2^)	1.096 (0.102)	1.072 (0.167)	*0.865 (0.055)*	*0.902 (0.120)*
T-Score, femur total	0.67 (0.81)	0.52 (1.33)	*−0.83 (0.37)*	*−1.07 (0.35)*
Z-Score, femur total	0.66 (0.37) *	**0.11 (0.26)**	0.47 (0.31) *	***0.15 (0.18)***

BMD: bone mineral density; *: significant difference between former gymnasts and controls for a given sex, Student’s *t*-test assuming equal variances (*p* < 0.05). Significant differences are highlighted in bold type.

**Table 4:** Characteristics of former gymnasts and reference populations in Brazil and Portugal–mean (standard deviation).

Variable	Males			*Females*		
	Former Gymnasts (n = 32)	Brazil (n = 354)	Portugal (n = 528)	*Former Gymnasts* *(n = 33)*	*Brazil* *(n = 474)*	*Portugal* *(n = 561)*
**General characteristics**						
Age (year)	60 (9)	60 (12)	57 (9)	*55 (8)*	64 (12) *	61 (9) *
Menopause onset (year)	-	-	-	*49 (2), n = 26*	***-***	*50 (4), n = 457*
Years after menopause onset (year)	-	-	-	*8 (8), range 1–27*	***-***	*15 (8), range 1–37*
Body mass (kg)	78.1 (11.5)	79.1 (14.6)	83.4 (16.6) *	*61.4 (9.3)*	68.9 (14.6) *	69.9 (15.5) *
BMI (kg/m^2^)	27.3 (3.9)	27.0 (4.3)	29.5 (15.2) *	*24.8 (3.6)*	27.8 (5.4) *	29.0 (6.1) *
Overweight/obesity n (%)	22 (69)	230 (65)	413 (78) *	*12 (36)*	***322 (68) ^a^***	404 (72) *
**Bone densitometry**						
BMC whole-body total (g)	2920.5 (324.6)	2791.6 (441.8) *	2488.9 (401.4) *	*2118.4 (238.7)*	2043.1 (365.2) *	1865.5 (315.8) *
BMD whole-body (g/cm^2^)	1.312 (0.130)	1.173 (0.140) *	1.144 (0.112) *	*1.108 (0.794)*	1.034 (0.135) *	1.031 (0.103) *
T-Score, whole-body	1.10 (1.29)	−0.26 (1.39) *	−0.61 (1.21) *	*0.14 (0.89)*	−0.48 (1.33) *	−1.00 (1.33) *
Z-Score, whole-body	1.28 (1.14)	−0.17 (1.22) *	−0.28 (1.03) *	*0.84 (0.93)*	0.33 (1.05) *	−0.21 (1.01) *
BMD femur neck (g/cm^2^)	1.996 (0.106)	1.128 (0.199) *	0.821 (0.133) *	*0.994 (0.151)*	*1.073 (0.198)*	0.731 (0.126) *
T-Score, femur neck	−0.08 (0.65)	−0.46 (1.44) *	−0.80 (0.99) *	*−0.62 (0.91)*	*−0.67 (1.36)*	−1.07 (1.14) *
Z-Score, femur neck	0.64 (0.66)	−0.29 (1.37) *	0.10 (0.97) *	*0.48 (0.94)*	−0.01 (1.25) *	*0.35 (1.04)*
BMD femur total (g/cm^2^)	1.084 (0.112)	1.054 (0.153) *	0.989 (0.142) *	*0.952 (0.140)*	*0.984 (0.142)*	0.864 (0.129) *
T-Score, femur total	0.56 (0.79)	−0.20 (1.13) *	−0.29 (0.95) *	*−0.28 (1.05)*	*−0.21 (1.13)*	−0.64 (1.06) *
Z-Score, femur total	0.47 (0.72)	−0.11 (1.05) *	0.16 (0.90) *	*0.64 (0.89)*	0.26 (1.08) *	0.49 (1.01) *
**Incidence of osteopenia and** **osteoporosis**						
Osteopenia n (%)	1 (3)	**96 (27) ^a^**	**202 (38) ^a^**	*11 (33)*	*183 (39)*	***244 (44) ^a^***
Osteoporosis n (%)	0 (0)	**30 (9) ^a^**	**33 (6) ^a^**	*0 (0)*	***57 (12) ^a^***	***48 (9) ^a^***

BMI: body mass index; BMD: bone mineral density. *: significant difference between former gymnasts and the reference population for a given sex, Student’s *t*-test not assuming equal variances (*p* < 0.05); ^a^: significant difference between former gymnasts and reference population for a given sex, chi-square test (*p* < 0.05). Significant differences are highlighted in bold type.

**Table 5:** Characteristics of former gymnasts and reference populations in Brazil and Portugal stratified by age subgroups (<60 years and ≥60 years)—mean (standard deviation).

Variable	Age < 60 Years					
	Males			*Females*		
	Former Gymnasts (n = 16)	Brazil (n = 204)	Portugal (n = 319)	*Former Gymnasts* *(n = 26)*	*Brazil* *(n = 202)*	*Portugal* *(n = 225)*
**General characteristics**						
Age (year)	54 (4)	52 (4)	51 (4) *	*52 (4)*	*52 (4)*	*53 (5)*
Menopause onset (year)	-	-	-	*49 (1), n = 19*	***-***	*50 (4), n = 90*
Years after menopause onset (year)	-	-	-	*4 (4), range 1–11*	***-***	*6 (4), range 1–22*
Body mass (kg)	80.3 (10.4)	82.0 (14.9)	86.3 (17.7) *	*61.2 (9.8)*	71.7 (14.4) *	75.3 (19.1) *
BMI (kg/m^2^)	27.8 (3.9)	27.5 (4.6)	29.3 (10.8)	*24.5 (3.8)*	27.7 (5.3) *	30.3 (7.7) *
Overweight/obesity n (%)	14 (88)	136 (67)	242 (76)	*7 (27)*	***140 (69) ^a^***	***162 (72) ^a^***
**Bone densitometry**						
BMC whole-body total (g)	2914.8 (345.3)	2883.9 (412.2)	2577.1 (401.2) *	*2155.2 (238.6)*	*2247.2 (328.9)*	1979.5 (319.8) *
BMD whole-body (g/cm^2^)	1.295 (0.142)	1.196 (0.130) *	1.159 (0.109) *	*1.125 (0.075)*	1.106 (0.111) *	1.057 (0.099) *
T-Score, whole-body	0.94 (1.40)	−0.05 (1.29) *	−0.45 (1.20) *	*0.31 (0.87)*	0.23 (1.12) *	−0.67 (1.25) *
Z-Score, whole-body	0.90 (1.11)	−0.22 (1.19) *	−0.26 (1.02) *	*0.81 (0.99)*	0.39 (1.07) *	−0.37 (0.98) *
BMD femur neck (g/cm^2^)	1.004 (0.117)	1.131 (0.187) *	0.838 (0.138) *	*0.912 (0.160)*	*1.109 (0.187)*	0.788 (0.127) *
T-Score, femur neck	0.08 (0.62)	−0.51 (1.34) *	−0.68 (1.02) *	*−0.43 (0.90)*	*−0.45 (1.32)*	−0.55 (1.15) *
Z-Score, femur neck	0.65 (0.57)	−0.39 (1.28) *	0.07 (0.98) *	*0.52 (0.95)*	−0.02 (1.27) *	*0.33 (1.11)*
BMD femur total (g/cm^2^)	1.071 (0.123)	1.057 (0.158) *	0.998 (0.148) *	*0.976 (0.147)*	*0.997 (1.136)*	0.909 (0.124) *
T-Score, femur total	0.45 (0.98)	−0.22 (1.12) *	−0.23 (0.98) *	*−0.07 (1.06)*	−0.10 (1.08) *	−0.27 (1.01) *
Z-Score, femur total	0.28 (0.65)	−0.11 (1.08) *	0.09 (0.67) *	*0.58 (1.10)*	0.22 (0.85) *	*0.31 (0.99)*
**Incidence of osteopenia and** **osteoporosis**						
Osteopenia n (%)	0 (0)	**51 (25) ^a^**	**102 (33) ^a^**	*7 (27)*	*61 (30)*	***68 (36) ^a^***
Osteoporosis n (%)	0 (0)	**16 (8) ^a^**	**10 (4) ^a^**	*0 (0)*	***22 (5) ^a^***	*4 (2)*
**Variable**	**Age ≥ 60 Years**					
	**Males**			***Females***		
	**Former Gymnasts** **(n = 16)**	**Brazil** **(n = 150)**	**Portugal** **(n = 209)**	***Former Gymnasts*** ***(n = 7)***	***Brazil*** ***(n = 272)***	***Portugal*** ***(n = 336)***
**General characteristics**						
Age (year)	66 (7)	72 (8) *	68 (6)	*68 (6)*	74 (8) *	*69 (6)*
Menopause onset (year)	-	-	-	*49 (4), n = 7*	*-*	*50 (4), n = 336*
Years after menopause onset (year)	-	-	-	*19 (7), range 8–27*	*-*	*19 (6), range 1–37*
Body mass (kg)	75.9 (12.5)	75.2 (13.1)	78.9 (13.7)	*62.0 (8.2)*	*66.8 (14.5)*	*67.7 (12.5)*
BMI (kg/m^2^)	26.8 (4.1)	26.3 (3.7)	29.3 (17.9)	*25.7 (3.1)*	*27.9 (5.5)*	*28.7 (4.9)*
Overweight/obesity n (%)	8 (50)	94 (63)	168 (80) *	*5 (71)*	*182 (77)*	*253 (75)*
**Bone densitometry**						
BMC whole-body total (g)	2926.2 (313.7)	2666.2 (451.2) *	2362.0 (367.0) *	*1981.6 (197.4)*	*1891.4 (313.7)*	1777.2 (283.3) *
BMD whole-body (g/cm^2^)	1.329 (0.120)	1.142 (0.147) *	1.121 (0.113) *	*1.047 (0.065)*	0.980 (0.125) *	*1.012 (0.101)*
T-Score, whole-body	1.26 (1.17)	−0.56 (0.96) *	−0.83 (0.98) *	*−0.49 (0.67)*	−1.10 (0.98) *	−1.25 (0.93) *
Z-Score, whole-body	1.68 (0.56)	−0.10 (0.97) *	−0.32 (1.04) *	*0.96 (0.49)*	0.28 (1.03) *	−0.09 (1.01) *
BMD femur neck (g/cm^2^)	0.988 (0.097)	1.121 (0.234) *	0.797 (0.123) *	*0.824 (0.084)*	*0.984 (0.200)*	*0.698 (0.113)*
T-Score, femur neck	−0.24 (0.66)	−0.33 (1.70)	−0.98 (0.81) *	*−1.34 (0.44)*	*−1.17 (1.33)*	*−1.34 (1.01)*
Z-Score, femur neck	0.65 (0.36)	0.02 (1.57) *	0.15 (0.88) *	*0.33 (0.44)*	0.01 (1.23) *	*0.36 (0.99)*
BMD femur total (g/cm^2^)	1.096 (0.102)	1.045 (0.144)	0.965 (0.134) *	*0.865 (0.055)*	0.949 (0.153) *	*0.839 (0.125)*
T-Score, femur total	0.67 (0.81)	−0.16 (1.15) *	−0.38 (0.79) *	*−0.83 (0.37)*	−0.48 (1.22) *	*−0.85 (0.97)*
Z-Score, femur total	0.66 (0.37)	−0.09 (0.93) *	0.23 (0.71) *	*0.47 (0.31)*	*0.37 (1.16)*	*0.59 (1.01)*
**Incidence of osteopenia and** **osteoporosis**						
Osteopenia n (%)	1 (6)	**45 (30) ^a^**	**100 (48) ^a^**	*4 (57)*	*122 (45)*	*176 (52)*
Osteoporosis n (%)	0 (0)	**14 (9) ^a^**	**23 (11) ^a^**	*0 (0)*	***35 (13) ^a^***	***44 (13) ^a^***

BMI: body mass index; BMD: bone mineral density. *: significant difference between former gymnasts and the reference population for a given sex, Student’s *t*-test not assuming equal variances (*p* < 0.05); ^a^: significant difference between former gymnasts and reference population for a given sex, chi-square test (*p* < 0.05). Significant differences are highlighted in bold type.

## Data Availability

The raw data supporting the conclusions of this article will be made available by the authors upon request.

## Supplementary Materials

The following supporting information can be downloaded at: https://www.mdpi.com/article/10.3390/ijerph23020159/s1, Table S1: Characteristics of former gymnast samples in Brazil and Portugal—mean (standard deviation), Table S2: Major physical activity performed in the last decade (PA-10) and during youth (PA-Youth) by former gymnasts (n = 65) and controls (n = 91) of both sexes, Table S3: Cohen’s d values and statistical power for comparisons between former gymnasts and controls, Table S4: Partial eta squared (ηp^2^) values and statistical power for ANCOVA comparisons between former gymnasts and controls (covariates age and physical activity in the last 10 years, Table S5. Bone densitometry variables in postmenopausal females (former gymnasts and controls) by hormone replacement therapy and calcium supplementation (mean ± SD), Table S6. Cohen’s d values and statistical power for comparisons between former gymnasts and reference populations in Brazil (BR) and Portugal (PT).

## Abbreviations

The following abbreviations are used in this manuscript:

AG	Artistic Gymnastics
ANCOVA	Analysis of Covariance
ASM	Appendicular Skeletal Muscle Mass
BMC	Bone Mineral Content
BMD	Bone Mineral Density
BMI	Body Mass Index
CIAFEL	Research Center for Physical Activity, Health, and Leisure
DXA	Dual Energy X-ray Absorptiometry
FIBRA	Frailty in Brazilian Older Adults
LABSAU	Laboratory of Physical Activity and Health Promotion
NHANES	National Health and Nutrition Examination Survey
PA-Youth	Physical activity volume during youth (10-20 years)
PA-10	Physical activity volume in the past 10 years
