# Supplementary material for: Bone Health in Former Artistic Gymnasts Aged 45 Years and Over: Case–Control Comparison with Controls and Reference Populations
Source: Int J Environ Res Public Health. 2026 Jan 27;23(2):159. doi: 10.3390/ijerph23020159 (PMC12941258; doi:10.3390/ijerph23020159)
Supplement: Supplementary file 1 [file ijerph-23-00159-s001.zip › ijerph-4058381-supplementary.pdf]

Supplementary Material

Table S1. Characteristics of former gymnast samples in Brazil and Portugal – mean (standard deviation).

Variable	Males		*Females*	
	**Brazil (n = 23)**	**Portugal (n = 9)**	***Brazil (n = 22)***	***Portugal (n = 11)***
**General**				
Age (y)	**62 (9)** [*]	**55 (7)**	*56 (8)*	*53 (8)*
Height (m)	1.69 (0.05)	1.70 (0.06)	*1.57 (0.05)*	*1.59 (0.04)*
Body mass (kg)	78.6 (13.1)	76.9 (6.7)	*60.1 (8.2)*	*63.1 (11.2)*
BMI (kg/m²)	27.6 (4.4)	26.6 (2.2)	*24.4 (3.0)*	*25.0 (4.6)*
Menopause onset (y)	-	-	*49 (3), n=19*	*50 (1), n=7*
Years after menopause onset (y)	-	-	*8.2 (7.9)*	*5.1 (9.3)*
PA-Youth (points)	11,600 (3,795)	8,883 (3,705)	*12,197 (6,716)*	*10,503 (3,466)*
PA-10 (points)	1,225 (1.292)	658 (617)	*820 (915)*	*886 (903)*
**Artistics Gymnastics (AG)**				
Age of AG beginning (y)	11 (3)	7 (4)	*8 (2)*	*7 (3)*
Time of AG training (y)	16 (5)	13 (5)	*11 (4)*	*12 (3)*
Training frequency (d/wk)	6 (1)	5 (1)	*6 (1)*	*6 (0)*
Training volume (h/day)	5 (1)	4 (0)	*6 (2)*	*5 (1)*
**Chronic Disease**				
Diabetes (n)	2	-	-	-
Hypertension (n)	8	1	*1*	-
Hypo/Hyperthyroidism (n)	1	-	*4*	-
Dyslipidemia (n)	1	-	*2*	*1*
Cervical/Lumbar pain (n)	2	-	-	-
Chronic pain (n)	-	-	-	*1*
Cardiac disease (n)	-	1	-	-
Respiratory disease (n)	-	-	-	*2*
Overweight/obesity (n)	**16** [a]	**6**	***8*** [a]	**3**
**Bone densitometry**				
BMC whole body (g)	3,013.0 (296,4)	2,684.2 (281.3)	*2,166.0 (235.5)*	*2,023.3 (225.8)*
BMD whole body (g/cm²)	1.356 (0.114)	1.200 (0.102)	*1.126 (0.083)*	*1.072 (0.060)*
Z-Score, whole body	1.70 (0.92)	1.11 (0.95)	*1.34 (0.62)*	*1.06 (0.59)*
BMD femur neck (g/cm²)	1.030 (0.096)	0.911 (0.084)	*0.950 (0.128)*	*0.780 (0.129)*
T-Score, femur neck	-0.06 (0.68)	-0.14 (0.62)	*-0.61 (0.92)*	*-0.64 (1.16)*
Z-Score, femur neck	0.60 (0.58)	0.73 (0.64)	*0.57 (0.84)*	*0.29 (1.15)*
BMD femur total (g/cm²)	1.114 (0.099)	1.005 (0.108)	*0.996 (0.119)*	*0.865 (0.142)*
T-Score, femur total	0.85 (0.79)	0.19 (0.70)	*-0.10 (0.95)*	*-0.64 (1.17)*
Z-Score, femur total	0.57 (0.70)	0.20 (0.76)	*0.81 (0.77)*	*-0.01 (1.18)*
**Body composition**				
Fat mass (g)	21,815 (9,005)	17,085 (4,115)	*20,414 (6,949)*	*20,481 (8,041)*
Relative fat (%)	28.1 (7.9)	22.2 (4.5)	*34.5 (7.5)*	*31.8 (7.2)*
Lean mass (g)	53,561 (5,962)	56,857 (4,913)	*37,266 (3,373)*	*40,160 (4,386)*
ASM/Height² (kg/m²)	8.58 (1.12)	8.84 (0.79)	*6.68 (0.59)*	*6.67 (0.87)*

BMI: body mass index; PA-Youth: physical activity volume in youth; PA-10: physical activity volume in the past 10 years; BMC: bone mineral content; BMD: areal bone mineral density; ASM: appendicular skeletal muscle mass. *: difference between Brazil and Portugal for a given sex, Student's t-test ($p<0.05$); a: difference between Brazil and Portugal for a given sex, chi-square test ($p<0.05$). Significant differences are highlighted in bold type.

Table S2. Major physical activity performed in the last decade (PA-10) and during youth (PA-Youth) by former gymnasts (n = 65) and controls (n = 91) of both sexes.

Activity	PA-10				PA-Youth			
	Former gymnasts		Controls		Former gymnasts		Controls	
	Males (n = 32)	*Females (n = 33)*	*Males (n = 37)*	*Females (n = 54)*	*Males (n = 32)*	*Females (n = 33)*	*Males (n = 37)*	*Females (n = 54)*
No activity	2	8	5	5			3	24
Aerobics/Callisthenics		1		2				3
American football			1					
Artistic gymnastics *	2	2			30	25		2
Ballet/Dance		3		2		3		6
Basketball					1			1
Capoeira							2	
CrossFit	2	1		1				
Cycling	3	2	2	1				2
Functional training				1				
Futsal		1						
Golf	1							
Handball							1	
Hawaiian canoeing		1						
Judo/Jiu-Jitsu							3	1
Karate							1	1
Physical education							1	1
Pilates	1	1	1	6				
Rowing							1	
Sailing		1				1		
Soccer			2				11	1
Strength training	8	5	14	18			2	2
Surfing/Kitesurfing	2						2	
Swimming/Aquatic exercise	2		2	6	1		2	5
Synchronized swimming						1		
Tennis	1			1				
Track and field							2	
Trampoline/Tumbling						3		
Volleyball	1			1			2	3
Walking/Running	9	4	9	9			4	2
Wrestling			1					
Yoga		3		1				

*: All former gymnasts participated in artistic gymnastics systematic training during youth. The frequencies of PA-Youth in this group represent additional activities performed besides gymnastics training, except for 'artistic gymnastics' which frequencies indicate participants who did not engage in any other activity. In the case of PA-10, frequencies refer to recreational and not competitive gymnastics.

Table S3. Cohen's d values and statistical power for comparisons between former gymnasts and controls. Cohen's d values are interpreted as small (≈0.2), moderate (≈0.5), and large (≥0.8).

Variable	**Males (d)**	**Males (Power, (1–β)**	***Females (d)***	***Females (Power, (1–β)***
BMC whole body (g)	-0.06	0.06	*0.21*	*0.19*
BMD whole body (g/cm²)	0.07	0.07	*0.13*	*0.12*
Z-Score, whole body	0.46	0.49	*0.1*	*0.09*
BMD femur neck (g/cm²)	-0.19	0.15	*0.01*	*0.06*
T-Score, femur neck	0.13	0.11	*0.68*	*0.94*
Z-Score, femur neck	0.56	0.67	*0.79*	*0.99*
BMD femur total (g/cm²)	0.13	0.12	*0.14*	*0.13*
T-Score, femur total	0.09	0.08	*0.33*	*0.37*
Z-Score, femur total	0.56	0.68	*0.46*	*0.54*

BMC: bone mineral content; BMD: bone mineral density.

Table S4. Partial eta squared (ηp^2) values and statistical power for ANCOVA comparisons between former gymnasts and controls (covariates age and physical activity in the last 10 years. Cohen's d values are interpreted as small (≈0.10), moderate (≈0.25), and large (≥0.40).

Variable	**Males – ηp^2**	**Males – Power (1–β)**	***Females – ηp^2***	***Females – Power (1–β)***
BMD whole body (g)	0.0	0.05	*0.002*	*0.06*
Z-Score whole body	0.028	0.32	*0.0*	*0.05*
BMD femur neck (g/cm²)	0.006	0.11	*0.014*	*0.18*
T-Score femur neck	0.004	0.09	*0.078*	*0.48*
Z-Score femur neck	0.102	0.67	*0.084*	*0.53*
BMD femur total (g/cm²)	0.005	0.1	*0.004*	*0.09*
T-Score femur total	0.003	0.08	*0.001*	*0.06*
Z-Score femur total	0.126	0.72	*0.09*	*0.55*

BMC: bone mineral content; BMD: bone mineral density.

Table S5. Bone densitometry variables in postmenopausal females (former gymnasts and controls) by hormone replacement therapy and calcium supplementation (mean ± SD).

Variable	**Replacement**		***No Replacement***	
	Former Gymnasts (n = 9)	Controls (n = 7)	*Former Gymnasts (n = 17)*	*Controls (n = 43)*
Age (years)	60 (8)	61 (8)	***55 (8)*** #	***61 (9)*** #
Menopause onset (y)	49 (2)	48 (4)	*49 (3)*	*48 (5)*
Time of menopause (y)	11 (9)	13 (11)	***6 (8)*** #	***13 (11)*** #
BMD whole body (g/cm²)	1.101 (0.075)	1.111 (0.106)	*1.085 (0.069)*	*1.091 (0.110)*
Z-Score, whole body	**1.34 (0.58)** *	1.07 (0.77)	***0.48 (0.84)*** *	*0.73 (1.01)*
BMD femur neck (g/cm²)	0.907 (0.087)	0.870 (0.124)	*0.855 (0.175)*	*0.888 (0.118)*
T-Score, femur neck	-0.90 (0.60)	-1.20 (0.89)	*-0.87 (0.98)*	*-1.06 (0.85)*
Z-Score, femur neck	**0.47 (0.47)** #	**-0.06 (0.49)** #	***0.31 (1.02)*** #	***-0.00 (0.81)*** #
BMD femur total (g/cm²)	0.964 (0.101)	0.887 (0.103)	*0.907 (0.151)*	*0.938 (0.115)*
T-Score, femur total	-0.37 (0.79)	-0.96 (0.80)	*-0.61 (1.06)*	*-0.55 (0.92)*
Z-Score, femur total	**0.79 (0.63)** #	**-0.13 (0.45)** #	*0.15 (1.04)*	*0.21 (0.93)*

BMD, bone mineral density; *: significant intergroup difference (replacement *vs. no replacement*); #: significant difference between gymnasts and controls within the replacement strata. Data were analyzed by one-way ANOVA followed by Fisher's post hoc test, equal variances not assumed ($p < 0.05$). Significant differences are highlighted in bold type.

Table S6. Cohen's d values and statistical power for comparisons between former gymnasts and reference populations in Brazil (BR) and Portugal (PT). Cohen's d values are interpreted as small (≈0.2), moderate (≈0.5), and large (≥0.8).

Variable	Sex	Population	Cohen's d	Power (1–β)
BMC whole body (g)	Males	BR	0.333	0.435
BMC whole body (g)	Males	PT	1.182	1.000
BMC whole body (g)	*Females*	*BR*	*0.244*	*0.272*
BMC whole body (g)	*Females*	*PT*	*0.903*	*0.999*
BMD whole body (g/cm^2)	Males	BR	1.029	1.000
BMD whole body (g/cm^2)	Males	PT	1.385	1.000
BMD whole body (g/cm^2)	*Females*	*BR*	*0.130*	*0.111*
BMD whole body (g/cm^2)	*Females*	*PT*	*0.136*	*0.118*
Z-Score whole body	Males	BR	1.228	1.000
Z-Score whole body	Males	PT	1.436	1.000
Z-Score whole body	*Females*	*BR*	*0.514*	*0.813*
Z-Score whole body	*Females*	*PT*	*1.082*	*1.000*
T-Score whole body	Males	BR	1.014	1.000
T-Score whole body	Males	PT	1.367	1.000
T-Score whole body	*Females*	*BR*	*0.548*	*0.859*
T-Score whole body	*Females*	*PT*	*1.007*	*1.000*
BMD femur neck (g/cm^2)	Males	BR	-0.828	0.994
BMD femur neck (g/cm^2)	Males	PT	1.455	1.000
BMD femur neck (g/cm^2)	*Females*	*BR*	*-0.449*	*0.701*
BMD femur neck (g/cm^2)	*Females*	*PT*	*1.891*	*1.000*
T-Score femur neck	Males	BR	0.340	0.452
T-Score femur neck	Males	PT	0.860	0.997
T-Score femur neck	*Females*	*BR*	*0.043*	*0.057*
T-Score femur neck	*Females*	*PT*	*0.436*	*0.681*
Z-Score femur neck	Males	BR	0.865	0.997
Z-Score femur neck	Males	PT	0.651	0.946
Z-Score femur neck	*Females*	*BR*	*0.443*	*0.690*
Z-Score femur neck	*Females*	*PT*	*0.131*	*0.113*
BMD femur total (g/cm^2)	Males	BR	0.224	0.227
BMD femur total (g/cm^2)	Males	PT	0.743	0.983
BMD femur total (g/cm^2)	*Females*	*BR*	*-0.227*	*0.242*
BMD femur total (g/cm^2)	*Females*	*PT*	*0.654*	*0.954*
T-Score femur total	Males	BR	0.780	0.988
T-Score femur total	Males	PT	0.973	1.000
T-Score femur total	*Females*	*BR*	*-0.064*	*0.065*
T-Score femur total	*Females*	*PT*	*0.341*	*0.477*
Z-Score femur total	Males	BR	0.644	0.936
Z-Score femur total	Males	PT	0.380	0.550
Z-Score femur total	*Females*	*BR*	*0.384*	*0.567*
Z-Score femur total	*Females*	*PT*	*0.158*	*0.142*

BMC: bone mineral content; BMD: bone mineral density.
